# Critical Care Echocardiography as a Routine Procedure for the Detection and Early Treatment of Cardiac Pathologies

**DOI:** 10.3390/diagnostics10090671

**Published:** 2020-09-04

**Authors:** Stefan Schmidt, Jana-Katharina Dieks, Michael Quintel, Onnen Moerer

**Affiliations:** 1 Department of Anesthesiology, Emergency and Intensive Care Medicine, University Hospital Göttingen, Georg-August-University, Robert-Koch-Str. 40, 37075 Göttingen, Germany; stefan.schmidt@med.uni-jena.de (S.S.); mquintel@med.uni-goettingen.de (M.Q.); omoerer@med.uni-goettingen.de (O.M.); 2 Department of Pediatric Cardiology and Pediatric Intensive Care Medicine, Georg-August University, Robert-Koch-Str. 40, 37075 Göttingen, Germany

**Keywords:** critical care echocardiography, intensive care, critical care, transthoracic echocardiography, cardiac disease, transthoracic image quality

## Abstract

Transthoracic and transesophageal echocardiography are important investigations in the intensive care unit (ICU) to diagnose acute cardiac pathologies and assess the haemodynamic status. Recommendations for critical care echocardiography (CCE) have been published recently, but these still lack an evidence-based foundation. It is not known if performing transthoracic echocardiography (TTE) on a routine basis instead of only when required in acute cases is feasible or clinically useful. In this single-centre prospective observational study, we routinely performed TTE on 111 consecutive non-cardiological, non-cardiothoracic surgical ICU patients in two surgical ICUs in a tertiary care facility. Significant cardiac pathologies were detected in 82 (76.6%) and critical cardiac pathologies in 33 (30.8%) of the 107 patients. The most common critical cardiac pathologies were sPAP > 50 mmHg (19.63%), tricuspid annular plane systolic excursion ≤ 13 mm (9.4%), grade III diastolic dysfunction (8.4%), severe tricuspid valve insufficiency (5.6%) and left ventricular ejection fraction (LV-EF) ˂ 30% (4.7%). Some of the most commonly found cardiac pathologies are not well emphasised in current recommendations and training programs. We observed a progression of the cardiac pathologies previously described in 41 of the patients (91.1%). Patients with echocardiographic abnormalities had a significant survival disadvantage in the ICU. By performing CCE routinely, we observed the range and prevalence of cardiac pathologies that can be detected by echocardiography in critically ill patients. We recommend routine transthoracic CCE in ICU patients for early detection of cardiac pathologies and to help inform early intervention regimens, since cardiac conditions carry a significant survival disadvantage for the ICU patient.

## 1. Introduction

Transthoracic (TTE) and transesophageal (TEE) echocardiography are important investigations for many patients in the intensive care unit (ICU) to diagnose acute cardiac pathologies and assess the haemodynamic status [1]. Critical care echocardiography (CCE) is its own specialty, with three competence levels: basic, advanced and expert. A recent international consensus statement [2] details the recommended training standards.

The requirements for attaining advanced CCE status have not been scientifically validated. While measurements such as tissue Doppler imaging (TDI) of the peak systolic velocity of the tricuspid annulus (S’) and the rate of pressure rise in the right ventricle (dP/dt) are rated as essential skills [2], measurement of the vena contracta, pulsed-wave Doppler of the pulmonary veins and proximal isovelocity surface area (PISA) measurements are not required.

The spectrum of cardiac pathologies in intensive care patients has not been systematically studied. The first aim of our study was, therefore, to detect and document the existing cardiac pathologies in critically ill patients by expert CCE.

Acute emergencies for which CCE is useful have been definitively specified [3,4,5,6,7], but cardiac pathologies that progress more slowly or might deteriorate, or combinations of cardiac pathologies that are relevant in the intensive care setting, have not yet been studied. The second aim of our study was, therefore, to determine the potential preventive value of routine expert CCE in the critically ill but not acutely deteriorating patients. Our objective was furthermore to define diagnostic findings or combinations of findings by which intensive care unit (ICU) patients who might benefit the most from routine CCE examinations can be identified.

TEE is conventionally used to evaluate acute conditions in the ICU. TTE, on the other hand, has generally received less attention and may remain underused even in medical ICUs [8]. Although TEE is a safe procedure with a complication rate of 0.18% to 2.8% [9], it is an invasive procedure [10]. TTE, however, is non-invasive and provides superior views for some haemodynamic evaluations, such as measuring transvalvular flows through the aortic, pulmonary and tricuspid valves or valid measurements of systolic pulmonary artery pressure (sPAP). TEE has been preferred because, in earlier studies, the obtainable images were previously of higher quality than those with TTE [11,12,13,14,15]. Recent studies, however, have shown a marked improvement in image quality and general feasibility of performing complete examinations with TTE in ICU patients [16,17]. Since both TTE and TEE are mandatory parts of advanced CCE training [2], the third aim of our study was to assess the feasibility of TTE in intensive care patients by expert CCE.

## 2. Materials and Methods

### 2.1. Institutional Approval of the Study Protocol and Enrolment Period

This manuscript adheres to the applicable STROBE guidelines. The study was approved by our institution’s ethics committee (ethics proposal Universitaetsmedizin Goettingen [UMG] 11/12/13, 18 February 2014 day month year) and registered in the German Clinical Trials Register (DRKS00009746, 10 February 2016). Informed written consent for performing the echocardiographic investigations was obtained from the participating patients or their legal guardians. One hundred and eleven consecutive patients were recruited for the study. The data of four patients were excluded from analysis after the initial consent had been retracted.

### 2.2. Patient Population and Time of Echocardiography

Patients with a non-cardiological and non-cardiothoracic surgical admission diagnosis, and who had not undergone a cardiothoracic operation in the recent past, were eligible for the study. TTE and clinical data analysis were performed on day three after admission to the ICU, to exclude patients admitted for uncomplicated postoperative observation. The study was conducted in two surgical ICUs that were supervised by anaesthesiologists of the University Hospital Göttingen, a tertiary care facility. The patients who would normally be admitted to these ICUs include those with sepsis, acute respiratory distress syndrome (ARDS) or major trauma, or those admitted following thoracic or trauma surgery or neurosurgery.

### 2.3. Detection of Cardiac Pathologies by Electrocardiogram and Chest X-ray

We assessed the reliability of the attending intensivists in detecting cardiac pathologies without input from echocardiography. A 12-channel electrocardiogram (ECG) was recorded in all patients on admission, and printouts were given to the attending consultant (at least six years of experience) and the resident (at least three years of experience) for evaluation. They were then requested to describe any signs of cardiac abnormalities. Chest X-rays were obtained if clinically indicated and were reviewed by the resident and consultant, who were then asked to find signs of cardiac pathologies. The intensivists were asked for their opinion on the necessity of TTE, their clinical suspicion of significant or critical cardiac pathologies (see below for definitions) and any therapeutic measures that might be necessary (Table 1).

After having completed their assessments, both intensivists were informed of the echocardiographic findings. Recommendations for further treatment were offered, based on the echocardiogram, and findings were integrated with the patient’s current haemodynamic state. The consultant was given a detailed written report of the echocardiographic findings.

### 2.4. Echocardiography Technique and Grading Scale

Cardiac abnormalities were diagnosed by using the expert CCE protocol, using all standard and non-standard echocardiographic windows, according to the guidelines of the American Society of Echocardiography [18,19,20,21,22,23,24], which took between 45 and 90 min. If these were not applicable for certain parameters, then the guidelines of the European Society of Cardiovascular Imaging [25,26] were used. Whenever the guidelines were not applicable or threshold values were not defined, the echocardiographic data were given numerically. We compiled a comprehensive list of common and unusual cardiac abnormalities, and defined the relevant ones beforehand as either significant or critical (Table S1). Intracardiac hypovolemia was diagnosed in conjunction with ventricular collapse, papillary muscle kissing sign and elaborate signs, such as very small end-diastolic areas and velocity time integral variations in the left ventricular outflow tract [27]. A single expert CCE examiner performed all echocardiograms. This examiner was trained in cardiology, anaesthesiology and intensive care medicine, and was, therefore, able to integrate directly the findings into changes in therapeutic management. The adult and paediatric cardiology departments were consulted in the event of non-distinctive or ambiguous findings.

In order to provide a more comprehensive assessment of transthoracic image quality than previously possible [16], we designed and used a new 15-point scale (where echo images of higher quality are assigned a higher score on the scale) that contains qualitative and quantitative criteria (Table S2).

A General Electric (GE) Healthcare Vivid S5 machine equipped with a phased array adult 1.5–3.6 MHz sector probe was used for the study. All necessary Doppler features (CD, PW, CW, TDI), imaging modalities (2D, M-mode) and software features (PISA, etc.) were available. Images were stored digitally and analysed immediately after each examination.

### 2.5. Data Handling and Statistical Analysis

Echocardiographic findings, patient characteristics, questionnaires and other relevant data from patient records were entered manually into the database and validated individually. Patient characteristics are shown in Table S3. They were then pseudo-anonymised, digitised and processed further with Microsoft Excel (Redmond, WA, USA). Sigmaplot (version 12.5, Systat Software Inc., San Jose, CA, USA) and OriginPro (version 9.2, OriginLab Corporation, Northampton, MA, USA) were used for statistical analysis. We calculated descriptive statistics, sensitivity and specificity, positive and negative predictive values, positive and negative likelihood ratios, diagnostic ratios and the Youden Index for diagnostic decisions. Quantitative data were compared using the *t*-test for independent data. Associations of cardiac pathologies with the Simplified Acute Physiology Score (SAPS II), days in ICU and age were evaluated by regression analysis. Survival analysis was performed with the Kaplan–Meier method with curve/group comparison assessed by the log-rank test. A *p*-value < 0.05 was considered statistically significant.

## 3. Results

### 3.1. Patient Characteristics

Details of patient characteristics and whether they received vasopressor or inotropic support are given in Tables S3 and S4.

### 3.2. Transthoracic Image Quality

The TTE images in 82% of the patients had a score of nine or better on our TTE image quality scale (Figure S1). This indicates that we were able to use at least two standard imaging windows and that the images were of sufficient quality to be able to quantify parameters such as cardiac index and sPAP. This still underrates the true value of the transthoracic approach, as we were able to determine a visual left ventricular ejection fraction (LV-EF) in 97% of the patients. We obtained valid LV-EF values using the Simpson method in 70% and sPAP values in 90% of patients. Translating our new grading system into the old system of usable windows revealed a score of at least two for 83% of our examinations (Table S5).

### 3.3. Range and Prevalence of Significant and Critical Cardiac Pathologies

Table S1 contains the list of cardiac pathologies defined as significant or critical. The most common significant cardiac pathologies (Table 2) were severe hypovolaemia (38.3%), reduced LV-EF (28%), left atrial volume index (LAVI) of ≥34 mL/m^2^ (27.1%), grade II diastolic dysfunction (24.3%) and dilated right atrium (22.4%). The most common critical cardiac pathologies (Table 3) were sPAP > 50 mmHg (19.63%), tricuspid annular plane systolic excursion ≤ 13 mm (9.4%), grade III diastolic dysfunction (8.4%), severe tricuspid valve insufficiency (5.6%) and LV-EF ˂ 30% (4.7%). A total of 76.6% of the patients had at least some form of predefined cardiac pathology. Many patients had more than one pathology or a combination of both significant and critical pathologies (Table 4), and more than half of the patients had three or more pathologies (Table S6).

### 3.4. Prediction of Cardiac Pathologies in Intensive Care Unit (ICU) Patients

Correlations of ICU parameters, questionnaires (*n* = 107) and data of ECG (*n* = 87) and chest X-ray (*n* = 103) interpretation were tested in order to predict their value in identifying a subgroup of patients that would benefit the most from routine CCE. A regression analysis of the SAPS II score, number of days in intensive care and age with TTE-diagnosed cardiac pathologies revealed no clinically useful correlation (maximum *R* and *R*^2^ values of 0.36 and 0.13; Table S7).

Of the patients with cardiac abnormalities shown by echocardiography, the consultant intensivists accurately predicted whether the patient had any cardiac abnormality in 79.3% of cases, and the residents in 76.8% (Table 1). The prediction sensitivity for significant pathologies in an individual patient was 79.3 for the consultants and 76.8% for the residents. Prediction specificity for any significant cardiac abnormality found by echocardiogram was 40% for consultants, and 64% for residents. 

Prediction sensitivity for critical cardiac pathologies was 33.3% for the consultants and 39.4% for the residents, and prediction specificity was 87.8% for the consultants and 81.1% for the residents. Of the patients who had an echocardiographic abnormality, 60% had previously been judged by a consultant intensivist, and 50% by a resident intensivist, that a TTE was unnecessary. Of the patients for whom a TTE was considered absolutely necessary by a consultant, 41.7% had an echocardiographic abnormality, and by a resident, 40.8%.

The sensitivities, specificities, PPVs, NPVs, likelihood ratios, diagnostic odds ratios and the Youden indices for ECG (*n* = 87) and chest X-ray interpretation (*n* = 103) are shown in Table 5. PPV is the probability that patients thought by the doctors to have a cardiac abnormality have one proven by echo; NPV is the probability that patients thought not to have an abnormality by the doctors have one excluded by echo. Usage of cardiac medication prior to admission was associated with similar predictive values. 

The sensitivities and PPVs increased significantly to 87.8% and 84.7%, respectively, and specificities and NPVs to 91.7% and 94.6% respectively, if the resident and consultant conferred before coming to a conclusion.

### 3.5. Value of Routine Critical Care Echocardiography (CCE)

We compared the cardiac pathologies listed in the patients’ medical records with our TTE results. In 43% of the patients, pre-existing cardiac pathologies were described that could be demonstrated by echocardiography. We were only able to confirm the recorded diagnoses by our own echocardiographic imaging in 48.9% of the cases. In the remaining 51.1% of the patients, abnormalities were detected that were not consistent with the diagnoses recorded in the patient’s medical history. In 8.9% of the patients with known cardiac pathologies, routine CCE revealed no additional findings, but additional abnormalities were found in the remaining 91.1% (Table S8).

**Figure 1:**
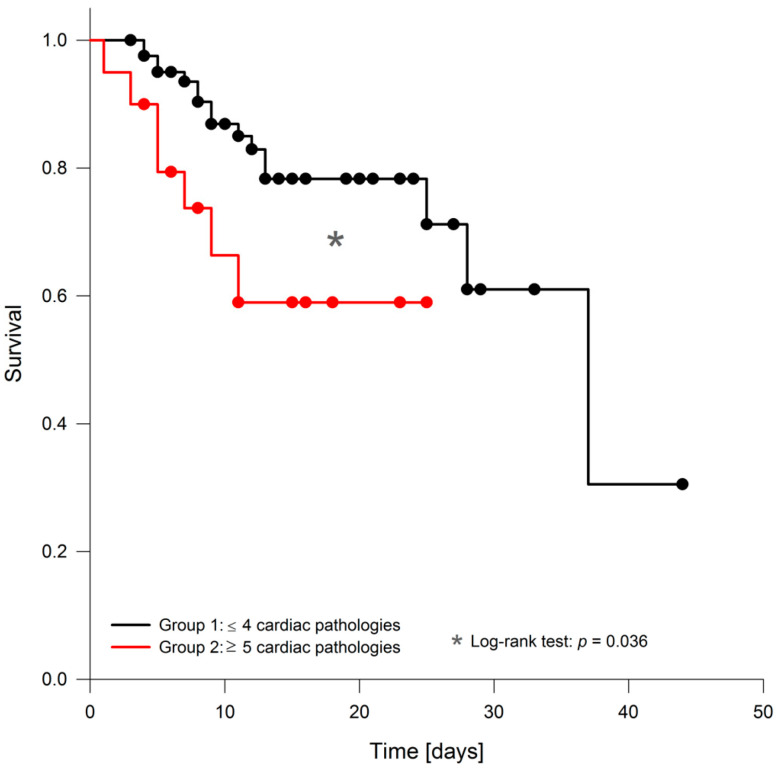
Kaplan–Meier survival curve. Kaplan–Meier survival curves of patients with four or fewer cardiac pathologies and patients with five or more cardiac pathologies.

Our study was not designed with survival as a primary endpoint. However, log-rank analysis of the Kaplan–Meier survival curves revealed a statistically significant survival disadvantage for patients with cardiac pathologies detected by echocardiography while in the ICU (Figure 1). Patients with four or fewer and patients with five or more cardiac pathologies had statistically significant mortality rates of 18.4% and 35%, respectively.

## 4. Discussion

Our study could meet all study aims outlined in the introduction. One major study aim was to assess the feasibility of TTE in intensive care patients. We showed that cardiac evaluation with TTE was successful in the majority of the patients despite difficulties such as obesity, restricted patient positioning and mechanical ventilation in some patients. In 99% of the patients, the TTE images yielded information relevant for treatment decisions. Measurements in the ICU that require highly defined 2D images (e.g., Simpson’s method for determining LV-EF) are less reliable than those using Doppler. Fortunately, the haemodynamic assessments obtained by Doppler echocardiography are far more important than precise LV-EF measurements. We, therefore, recommend TTE as the initial routine technique in the acute setting with a switch to TEE only if image quality is not satisfactory, if time is a vital issue or if the examiner is more skilled with TEE than with TTE.

Another major study aim was to define the range and prevalence of cardiac pathologies in ICU patients. The large percentage of patients found to have significant and/or critical cardiac pathologies emphasises the need for CCE to be performed routinely, instead of only when considered useful or necessary. In terms of significant pathologies, grade II diastolic dysfunction was nearly as common as a reduced LV-EF. Of the critical cardiac pathologies, severe right ventricular dysfunction and grade III diastolic dysfunction were about twice as common as severe left ventricular dysfunction. The results may have been influenced by the large number of patients with echocardiographic signs of hypovolemia, but this could be a common problem in the ICU [28,29] and does not reflect volume responsiveness [30]. Significant or critical heart valve disease was documented in more than a third of the patients. The PISA method and vena contracta measurements were indispensable for grading these heart valve dysfunctions, yet they are not a mandatory part of advanced CCE skills. Significant or critical increased sPAP together were present in two in five patients, and this might have contributed to haemodynamic instability, duration of mechanical ventilation and difficulty in weaning. Determination of sPAP from any suitable echocardiographic views should, therefore, be part of the routine assessment. Evaluation of artificial heart valves, not included in advanced CCE training, proved to be unnecessary as moderate or severe dysfunction of a prosthetic valve only occurred in 1.9% of examinations.

Parameters such as SAPS II score, days in intensive care or age cannot reliably identify patients with cardiac pathologies. Predicting a cardiac pathology based on clinical impression and knowledge of the patient had a PPV over 80% for both consultants and residents, but the NPV was low. This limits its clinical applicability. ECG and chest X-ray were also shown to be of limited value in the actual clinical situation, although they attained higher statistical values than previously reported [17].

Patient records and results from earlier diagnostic tests were available for approximately half of our patients. We were able to confirm about half of the documented prior diagnoses. Of the diagnoses we were unable to confirm, approximately half were implausible or unconfirmed (see details in legend to Table S8). In view of the large number of patients with underdiagnosed cardiac pathologies, re-examination should be mandatory in patients with known cardiac pathology. Based on our findings, we recommend routine CCE for every patient in intensive care. If this is not feasible for every patient, the ECG and the chest X-ray should be critically evaluated by two or more intensivists in collaboration to determine which patients might have cardiac pathologies. This approach yielded a PPV of 94.6% and an NPV of 52.4% in this study.

Although physical bedside evaluations like CVP waveforms, cardiac auscultation, cardiac percussion and arterial waveforms were indicative for some cardiac pathologies like reduced right ventricular ejection fraction and relevant tricuspid regurgitation, other cardiac pathologies like relevant diastolic dysfunction, reduced left ventricular function or increased systolic pulmonary artery pressure could not be reliably predicted. The presence of cardiac arrhythmia or the presence of multiple cardiac pathologies reduced the predictive value of physical bedside evaluations. The greatest strength of CCE in comparison to physical bedside evaluations was its quantitative nature.

The last major study aim was to assess the feasibility of TTE in intensive care patients by expert CCE. We were able to determine the potential preventive value of routine expert CCE in the critically ill but not acutely deteriorating patients. The mortality and survival analyses showed that the presence of cardiac pathologies almost doubled mortality rates and, thus, identified a subgroup of patients at particular risk. After completing the echocardiographic studies, the resident and consultant were informed of the cardiac pathologies, and treatment recommendations were immediately offered, but the survival disadvantage persisted even though the patients’ cardiac pathologies were known. Most therapy changes affected the choice of vasopressors or inotropes, fluid therapy regimens, pharmacological therapy, heart rate management and respirator settings. The combination of therapy changes was individualised to the specific patient based on CCE findings. However, the detrimental effect of cardiac pathologies on survival might have been even greater had the attending intensivists not been able to optimise treatment because they were unaware of the impaired cardiac function that was revealed by TTE.

Based on the most common cardiac pathologies found in our study, we recommend a minimum CCE protocol consisting of the evaluation of left and right ventricular functions (LV-EF, RV-EF, TAPSE); diastolic dysfunction (LAVI, E/e’); systolic artery pressure approximation (sPAP); cardiac dimensions; evaluation of the tricuspid, mitral and aortic valve; presence of pericardial effusions and signs of relevant hypovolemia (e.g.,maximal Doppler velocity in left ventricular outflow tract [∆VmaxAo]).

## 5. Limitations of the Study

The study has several potential limitations. A single expert CCE examiner performed all examinations. The study is limited in generalisability by the single-centre nature of the cohort. We attempted to offset this by performing it in two separate ICUs with eight different consultants who all followed slightly different treatment regimes. TTE was the only method employed, and we cannot exclude the possibility that TEE might have revealed additional pathologies in patients in whom TTE imaging quality was suboptimal. Statistically, the study results cannot be used to determine whether the use of routine CCE alters the patient outcome. Common sense favours this conclusion, but it remains an untested hypothesis.

## 6. Conclusions

We have demonstrated the feasibility of obtaining adequate image quality with transthoracic CCE, and have shown the benefit of expanding the use of CCE from a procedure performed only when considered useful, to a procedure performed routinely. In addition, we have determined the range and prevalence of cardiac pathologies in the ICU setting. We have shown that patients with cardiac pathologies are difficult to identify clinically and, therefore, we recommend routine CCE for all ICU patients. Finally, we have shown that patients with echocardiographic abnormalities have a higher mortality.

## Figures and Tables

**Table 1:** Questionnaire for consultants and residents regarding expected cardiac pathologies.

Questionnaire Answered by Consultants:								
I consider performing transthoracic echocardiography in this study participant as:								
not necessary	4.7%	less beneficial	15.9%	beneficial	57.9%	absolutely necessary	21.5%	
Results: Percentage of patients with cardiac pathologies in above category proven by echocardiography	60%		64.7%		77.4%		41.7%	
Do you expect a cardiac pathology in this study participant?								
	yes		74.8%		no		25.2%	
Results: Sensitivity	79.3%	Specificity	40%		PPV	81.3%	NPV	37%
Do you think that your expected cardiac pathology(ies) require special treatment while in the ICU?								
	yes		62.5 %		no		37.5%	
Do you expect your cardiac pathology(ies) to be a potentially critical cardiac pathology(ies)?								
	yes		25%		no		75%	
Results: Sensitivity	33.3%	Specificity	87.8%		PPV	55%	NPV	74.7%
**Questionnaire Answered by Residents:**								
I consider performing transthoracic echocardiography in this study participant as:								
not necessary	7.5%	less beneficial	15%	beneficial	54.2%	absolutely necessary	23.4%	
Results: Percentage of patients with cardiac pathologies in above category proven by echocardiography	50%		56.3%		84.5%		40.8%	
Do you expect a cardiac pathology in this study participant?								
	yes		67.3%		no		32.7%	
Results: Sensitivity	76.8%	Specificity	64%		PPV	87.5%	NPV	45.7%
Do you think that your expected cardiac pathology(ies) require special treatment while in the ICU?								
	yes		75%		no		25%	
Do you expect your cardiac pathology(ies) to be a potentially critical cardiac pathology(ies)?								
	yes		37.5%		no		62.5%	
Results: Sensitivity	39.4%	Specificity	81.1%		PPV	48.2%	NPV	75%

PPV: positive predictive value; NPV: negative predictive value; ICU: intensive care unit. Questionnaires/patients *n* = 107, assessed by eight different consultants and 21 residents.

**Table 2:** The ten most frequently observed significant cardiac pathologies.

1	Significant hypovolemia	38.3	%
2	Reduced left ventricular ejection fraction (LV-EF) (30–54.9%)	28	%
3	Left atrial volume index (LAVI) ≥ 34 mL/m^2^	27.1	%
4	Grade II diastolic dysfunction	24.3	%
5	Right atrium (RA) > 20 cm^2^	22.4	%
6	Regional wall motion abnormalities (RWMA)	21.5	%
7	Increased systolic pulmonary artery pressure (sPAP) (40–49.9 mmHg)	20.6	%
8	Left ventricular hypertrophy ≥ 14 mm	18.7	%
9	Pericardial effusion	11.2	%
10	Moderate tricuspid valve insufficiency	10.3	%

**Table 3:** The five most frequently observed critical cardiac pathologies.

1	Severely increased systolic pulmonary artery pressure (sPAP) (≥50 mmHg)	19.6	%
2	Severely reduced tricuspid annular plane systolic excursion (≤13 mm)	9.4	%
3	Grade III diastolic dysfuction	8.4	%
4	Severe tricuspid valve insufficiency	5.6	%
5a	Severely reduced left ventricular ejection fraction (LV-EF) (<30%)	4.7	%
5b	Severe right ventricular enlargement, defined as right ventricular diameter (RVD)1 ≥ 50 mm, RVD2 ≥ 46 mm or RVD3 ≥ 96 mm	4.7	%

**Table 4:** Incidence of cardiac abnormalities detected by expert critical care echocardiography (CCE).

No Significant or Critical Cardiac Abnormalities	Number of Patients with One or More Significant Cardiac Abnormalities on Echocardiogram	Number of Patients with One or More Critical Cardiac Abnormalities on Echocardiogram	Total Number of Significant and/or Critical Cardiac Abnormalities
25 (*n*) 23.4 (%)	80 (*n*) 74.8 (%)	33 (*n*) 30.8 (%)	82 (*n*) 76.6 (%)
number and percentage of patients			occurrence of multiple pathologies possible

**Table 5:** Electrocardiogram (ECG) and chest X-ray analysis.

Physician Rank	Diagnostic Test [Statistical Assumption]	Sensitivity (%)	Specificity (%)	PPV (%)	NPV (%)	LR+	LR-	DOR	Youden Index
Consultant	ECG	68.1	66.7	88.7	35.3	2.04	0.48	4.27	0.35
	Chest X-ray	67.1	70.8	88.3	39.5	2.30	0.46	4.95	0.38
	ECG and chest X-ray (believe the positives)	75.6	62.5	87.3	42.9	2.02	0.39	5.17	0.38
	ECG and chest X-ray (believe the negatives)	56.1	75.0	88.5	33.3	2.24	0.59	3.83	0.31
Resident	ECG	69.6	66.7	88.9	36.4	2.09	0.46	4.57	0.36
	Chest X-ray	64.6	62.5	85.0	34.9	1.72	0.57	3.04	0.27
	ECG and chest X-ray (believe the positives)	75.6	62.5	87.3	42.9	2.02	0.39	5.17	0.38
	ECG and chest X-ray (believe the negatives)	56.1	75.0	88.5	33.3	2.24	0.59	3.83	0.31
Pharmacotherapy	Chronic cardiac medication prior to hospital admission	64.6	68.0	86.9	37.0	2.02	0.52	3.88	0.33
Consultant and resident combined	ECG and chest X-ray (believe the positives)	87.8	45.8	84.7	52.4	1.62	0.27	6.09	0.34
	ECG and chest X-ray (believe the negatives)	42.7	91.7	94.6	31.9	5.12	0.63	8.19	0.34

Statistical values for ECG and/or chest X-ray analysis for the consultant and resident intensivist, and the presence of chronic cardiac medication prior to hospital admission is shown. Patients *n* = 107; ECGs *n* = 87; chest X-rays *n* = 103. PPV: positive predictive value; NPV: negative predictive value; LR+: positive likelihood ratio; LR-: negative likelihood ratio; DOR: diagnostic odds ratio. Believe the positives (statistical assumption) = either the consultant or the resident or both rated a positive finding either on ECG or on chest X-ray as suggestive of cardiac pathologies. Usually used for increasing the overall sensitivity. Believe the negatives (statistical assumption) = either the consultant or the resident or both rated a normal ECG and chest X-ray as non-suggestive of cardiac pathologies. Usually used for increasing the overall specificity.

## Supplementary Materials

The following are available online at https://www.mdpi.com/2075-4418/10/9/671/s1, Figure S1: Quality benchmark for grading quality of transthoracic critical care echocardiography. Table S1: Complete list of cardiac pathologies that were predefined as either “significant” or “critical”. Table S2: New objective scale for quantifying echocardiography image quality. Table S3: Patient characteristics. Table S4: Vasoactive drug therapy. Table S5: Alternative grading for transthoracic image quality. Table S6: Percentage of patients with significant and/or critical cardiac pathologies. Table S7: Correlations between cardiac pathologies and SAPS II score, days spent in intensive care and age. Table S8: Cardiac pathologies mentioned in discharge letters and available medical records.
