# Supplementary material for: Critical Care Echocardiography as a Routine Procedure for the Detection and Early Treatment of Cardiac Pathologies
Source: Diagnostics (Basel). 2020 Sep 4;10(9):671. doi: 10.3390/diagnostics10090671 (PMC7555963; doi:10.3390/diagnostics10090671)
Supplement: Supplementary file 1 [file diagnostics-10-00671-s001.pdf]

Figure S1 Quality benchmark for grading quality of transthoracic critical care echocardiography

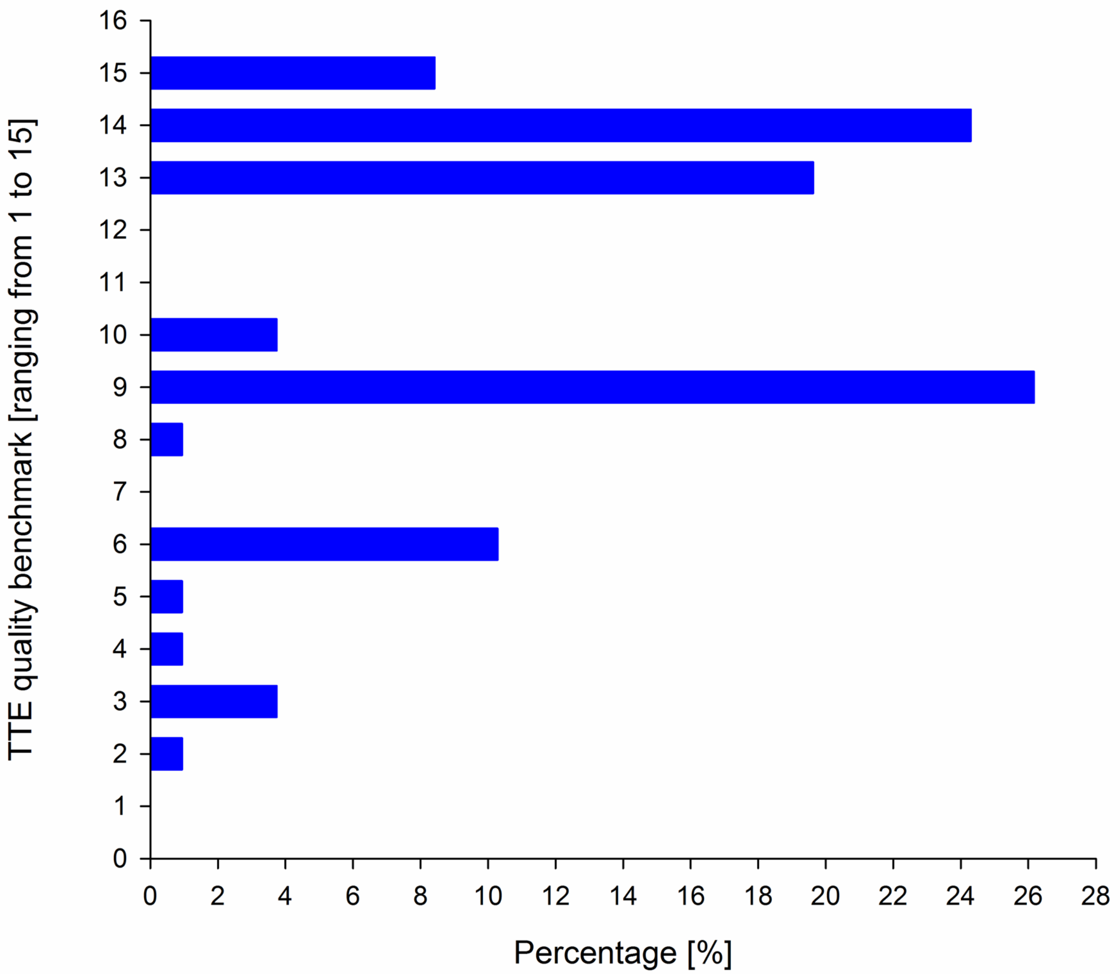

Table S1 Complete list of cardiac pathologies that were predefined as either “significant” or “critical”

**Predefined significant cardiac pathologies**	
Significant cardiac pathology	*[%]*
Reduced left ventricular ejection fraction (LV-EF) [30-54.9%]	28
Regional wall motion abnormalities (RWMA)	21.5
Left ventricular hypertrophy ≥14 mm	18.7
Moderate mitral valve insufficiency	8.4
Moderate mitral valve stenosis	0
Moderate aortic valve insufficiency	6.5
Moderate aortic valve stenosis	1.9
Moderate tricuspid valve insufficiency	10.3
Moderate tricuspid valve stenosis	0
Moderate pulmonary valve insufficiency	1.9
Moderate pulmonary valve stenosis	0
Reduced tricuspid annular plane systolic excursion [13.1-15.9 mm]	8.4
Right ventricular enlargement defined as right ventricular diameter (RVD)1 46-49 mm or RVD2 42-45 mm or RVD3 92-95 mm	3.7
Increased systolic pulmonary artery pressure (sPAP) [40-49.9 mmHg]	20.6
Pericardial effusion	11.2
Suspected intracardiac vegetation	0.9
Suspected intracardiac thrombus	0
Left ventricular diameter of 64-69 mm ♂ or 58-62 mm ♀	0.9
Left atrial volume index (LAVI) ≥34 ml/m²	27.1
Right atrium (RA) >20 cm²	22.4
Ascending aorta >40 mm	0
Grade II diastolic dysfunction	24.3
Suspected aortic dissection	0
Bicuspid aortic valve	0
Mitral valve prolapse	0.9
Intracardiac shunts	0
Moderate prosthetic valve dysfunction	0.9
Significant hypovolemia	38.3

Predefined critical cardiac pathologies

Critical cardiac pathology	*[%]*
Severely reduced left ventricular ejection fraction (LV-EF) [<30%]	4.7
Grade III diastolic dysfunction	8.4
Severely reduced tricuspid annular plane systolic excursion [≤13 mm]	9.4
Severe prosthetic valve dysfunction	0.9
Hypertrophic cardiomyopathy	0
Severely increased systolic pulmonary artery pressure (sPAP) [≥50 mmHg]	19.6
Severe mitral valve insufficiency	0
Severe mitral valve stenosis	0
Severe aortic valve insufficiency	0
Severe aortic valve stenosis	2.8
Severe tricuspid valve insufficiency	5.6
Severe tricuspid valve stenosis	0
Severe pulmonary valve insufficiency	0
Severe pulmonary valve stenosis	0
Left ventricular diameter of >62 mm ♀ or > 69mm ♂	0
Severe right ventricular enlargement defined as right ventricular diameter (RVD) 1 ≥50 mm or RVD2 ≥ 46mm or RVD3 ≥96 mm	4.7
Hemodynamically relevant pericardial effusion	0
Intracardiac vegetation	0
Intracardiac thrombus	0
Aortic dissection	0

Table S2 New objective scale for quantifying echocardiography image quality

1	No usable echocardiographic views
2	Only non-standardized echocardiographic views
3	Only one standardized echocardiographic view
4	At least one standardized echocardiographic view and at least one non-standardized echocardiographic view, quantifications not feasible
5	At least one standardized echocardiographic view and at least one non-standardized echocardiographic view, visual quantifications
6	At least one standardized echocardiographic view and at least one non-standardized echocardiographic view, limited objective quantifications
7	At least two standardized echocardiographic views, quantifications not feasible
8	At least two standardized echocardiographic views, visual quantifications
9	At least two standardized echocardiographic views, limited objective quantifications
10	At least two standardized echocardiographic views, objective quantifications
11	All standardized echocardiographic views, quantifications not feasible
12	All standardized echocardiographic views, visual quantifications
13	All standardized echocardiographic views, limited objective quantifications
14	All standardized echocardiographic views, objective quantifications
15	All standardized echocardiographic views, all cardiac structures depictable, all quantifications feasible, measurement of special parameters possible

A new, objective scale for quantifying transthoracic echocardiography image quality was developed using qualitative and quantitative factors to replace the existing grading tools that are largely subjective. The new grading tool slightly underrates the actual quality of obtained images and can be considered conservative. The images in 82% of patients had an image quality score of nine or higher, while clinically useful information such as the visually assessed LV-EF could be reported in 97% of patients. The new quantification system can describe examination quality more accurately than existing tools, that mostly only report the number of usable imaging windows.

Table S3 Patient characteristics

**Gender**	male	*73 (n) 68.2 (%)*	female	*34 (n) 31.8 (%)*
**Age [years]**	average	*67.4*	median	*68*
	min.	*20*	max.	*92*
**Body weight [kilograms]**	average	*81.5*	median	*80*
	min.	*45*	max.	*175*
**Body mass index [BMI]**	average	*27.1*	median	*26.0*
	min.	*19.4*	max.	*54*
**BMI > 30**		*19 (n) 17.8 (%)*		
**Days in intensive care**	average	*12.4*	median	*11*
**Outcome**	survived	*84 (n) 78.5 (%)*	died	*23 (n) 21.5 (%)*
**PiCCO®/PAC**	PiCCO®	*24 (n) 22.4 (%)*	PAC	*2 (n) 1.9 (%)*
**SAPS II score**	average	*33.3*	median	*33*
	min.	*12*	max.	*61*
**Mechanical ventilation**	yes	*73 (n) 68.2 (%)*	no	*34 (n) 31.8 (%)*
**Echocardiographic examination in**	left lateral position	*26 (n) 24.3 (%)*	supine position	*81 (n) 75.7 (%)*
**Chronic cardiac medication prior to hospital admission**	yes	*61 (n) 57 (%)*	no	*46 (n) 43 (%)*

Patient characteristics. PAC: pulmonary artery catheter; PiCCO®: pulse contour cardiac output system, Pulsion Medical Systems; Mean ± standard deviation (SD) unless otherwise stated.

Table S4 Vasoactive drug therapy

**Patients with vasoactive drug therapy**		
no 65 (n) 60.75 (%)	*yes*	*42 (n) 39.25 (%)*
**Type of applied catecholamine (multiple drugs possible)**		
Norepinephrine	*37 (n)*	*88.1 (%)*
Dobutamine	*13 (n)*	*40 (%)*
Epinephrine	*3 (n)*	*7.1 (%)*

Table S5 Alternative grading for transthoracic image quality

Number of imaging windows	Examinations
0	1 (n) 0.9 (%)
1	17 (n) 15.9 (%)
2	33 (n) 30.8 (%)
3	56 (n) 52.3 (%)

Grading transthoracic echocardiography image quality in our study patients employing a previously published approach of usable imaging windows to permit a comparison of our results with those published in the literature.

Table S6 Percentage of patients with significant and/or critical cardiac pathologies

**Percentage of patients with significant cardiac pathologies** *number of pathologies*							**Percentage of patients with critical cardiac pathologies** *number of pathologies*				
**1**	**2**	**3**	**4**	**5**	**6**	**7**	**1**	**2**	**3**	**4**	**5**
27.5	20	15	21.3	5	8.8	2.5	60.6	15.2	12.1	6.1	6.1
**Percentage of patients with significant and/or critical cardiac pathologies** *number of pathologies*											
**1**	**2**	**3**	**4**	**5**	**6**	**7**	**8**	**9**	**10**	**11**	**12**
23.2	18.3	13.4	20.7	7.3	4.9	2.4	2.4	3.7	1.2	1.2	1.2

Percentage of patients with given number of different cardiac pathologies.

Table S7 Correlations between cardiac pathologies and SAPS II score, days spent in intensive care and age

Correlation of SAPS II with significant cardiac pathologies	R = 0.362	R^2 = 0.131	adj. R^2 = 0.123
	Significant cardiac pathologies = 0.0176 + (0.0647 * SAPS II)		
Correlation of SAPS II with critical cardiac pathologies	R = 0.116	R^2 = 0.014	adj. R^2 = 0.004
	Critical cardiac pathologies = 0.162 + (0.0113 * SAPS II)		
Correlation of SAPS II with significant and critical cardiac pathologies	R = 0.310	R^2 = 0.096	adj. R^2 = 0.088
	Significant and critical cardiac pathologies = 0.179 + (0.0760 * SAPS II)		
Correlation of days spent in intensive care with significant and critical cardiac pathologies	R = 0.103	R^2= 0.011	adj. R^2 = 0.001
	Cardiac pathologies = 3.164 - (0.0336 * days in intensive care)		
Correlation of age with significant and critical cardiac pathologies	R = 0.318	R^2 = 0.101	adj. R^2 = 0.093
	Cardiac pathologies = -1.806 + (0.0675 * Age)		

R: correlation coefficient; R^2: coefficient of determination; adj. R^2: adjusted R-squared value.

Table S8 Cardiac pathologies mentioned in discharge letters and available medical records

**Were cardiac pathologies mentioned in the patient's medical record?**	*yes*	43 %	*no*	57 %
**Did routine CCE confirm the mentioned pathologies or were they at least plausible?**	*confirmed or plausible*	48.9 %	*unconfirmed or implausible*	51.1 %
**Were additional pathologies detected in patients who had at least one cardiac pathology mentioned in their medical records?**	*yes*	91.1 %	*no*	8.9 %

The large number of diagnoses beyond those actually detected might be influenced by the German billing system in which increasing the number of symptoms or examination findings can markedly increase the reimbursement by the health insurance company. Alternatively, these findings could be due to the lack of compulsory official training programmes or assessments for TTE in internal medicine in the German medical system. We noticed that a high proportion of diagnoses from smaller hospitals or hospitals that had no dedicated cardiology department were inaccurate, while no report from a university hospital was inaccurate. We personally interpret the high degree of additional cardiac pathologies in patients with previously known cardiac pathologies to be due to the rapid changes in cardiac function and dimensions in the course of severe illness and intensive care. However, these pathologies might well have been overlooked or not reported originally.
